# QTL mapping for Mediterranean corn borer resistance in European flint germplasm using recombinant inbred lines

**DOI:** 10.1186/1471-2164-11-174

**Published:** 2010-03-15

**Authors:** Bernardo Ordas, Rosa A Malvar, Rogelio Santiago, Ana Butron

**Affiliations:** 1 Misión Biológica de Galicia, Spanish National Research Council (CSIC). Apartado 28, 36080 Pontevedra, Spain

## Abstract

**Background:**

*Ostrinia nubilalis *(ECB) and *Sesamia nonagrioides *(MCB) are two maize stem borers which cause important losses in temperate maize production, but QTL analyses for corn borer resistance were mostly restricted to ECB resistance and maize materials genetically related (mapping populations derived from B73). Therefore, the objective of this work was to identify and characterize QTLs for MCB resistance and agronomic traits in a RILs population derived from European flint inbreds.

**Results:**

Three QTLs were detected for stalk tunnel length at bins 1.02, 3.05 and 8.05 which explained 7.5% of the RILs genotypic variance. The QTL at bin 3.05 was co-located to a QTL related to plant height and grain humidity and the QTL at bin 8.05 was located near a QTL related to yield.

**Conclusions:**

Our results, when compared with results from other authors, suggest the presence of genes involved in cell wall biosynthesis or fortification with effects on resistance to different corn borer species and digestibility for dairy cattle. Particularly, we proposed five candidate genes related to cell wall characteristics which could explain the QTL for stalk tunnelling in the region 3.05. However, the small proportion of genotypic variance explained by the QTLs suggest that there are also many other genes of small effect regulating MCB resistance and we conclude that MAS seems not promising for this trait. Two QTLs detected for stalk tunnelling overlap with QTLs for agronomic traits, indicating the presence of pleitropism or linkage between genes affecting resistance and agronomic traits.

## Background

*Ostrinia nubilalis *(ECB) and *Sesamia nonagrioides *(MCB) are two maize stem borers which cause important losses in temperate maize production. ECB is present in United States and Central and South Europe, while *Sesamia nonagrioides *(MCB) is restricted to Mediterranean areas, including South Europe, North Africa and Middle East [1,2]. Larvae feed on the stem, producing tunnels that weaken the plant and, as consequence, stalk lodging is increased and yield reduced. Furthermore, larvae can also feed directly on the ear which promotes infections by *Fusarium *spp at levels that may affect human and animal health [3,4]. Although the type of damage caused by the two species is similar, MCB larvae are more voracious and produce more damage than ECB larvae [2]. Phenotypic evaluations for resistance to ECB and MCB suggest that maize has common mechanisms of resistance to both pests [5,6].

Several studies have been carried out to map genetic factors for resistance to ECB tunnelling [7-9], but only one QTL analysis for resistance to MCB tunnelling has been reported so far [10]. The two QTLs for MCB resistance detected were located close to QTLs for ECB resistance which could indicate the presence of gene clusters or common mechanisms of resistance to different pests. However, more QTL experiments for resistance to MCB are needed to confirm the co-localization of QTLs for resistance to both pests. The search for QTLs rather should be done with no previously prospected maize materials than with materials extensively studied such as those derived from B73 [7-10] in order to likely increase the number of known genomic regions involved in borer resistance, as, in general, different subsets of QTLs can segregate in different populations [11].

Significant variation for resistance to ECB and MCB was found in the European Union Maize Landrace Core collection and some populations from Central and Eastern Spain seemed to be promising sources of resistance to maize stem borers, that is, those populations probably carry favourable alleles for ECB or MCB resistance [5,12,13]. Consequently, the use of mapping populations derived from these European materials could allow to widen the already known genomic regions for corn borer resistance.

Additive effects have been consistently reported as more important than dominant effects for stalk tunnelling by MCB [14-16], while additive and dominant effects has been reported as important for MCB ear resistance and yield under infestation [17,18]. It is interesting to mention that stalk tunnel length, the character typically used to quantify corn borer damage, is negatively correlated with yield [19-21], although the genetic mechanism responsible of that relationship, whether pleitropy or repulsion linkage, is unknown. A population of recombinant inbred lines (RILs) is a useful tool for mapping QTLs for traits under additive control because RILs represent a permanent sample of progenies for evaluations using replications in different environments.

Most inbreds used in temperate zones derive from Corn Belt Dent varieties, but adapted flint lines derived from European populations are also widely used, particularly, in Europe, northern areas of North America, and Japan [22-25]. A heterotic pattern widely used by western European breeders is Corn Belt Dent × European Flint [23,26-28] using preferentially materials from the Still Stalk Synthetic because the Stiff Stalk Synthetic subgroup shows more heterosis with European Flint than other Corn Belt Dent subgroups [26]. A population of RILs derived from two European flint lines will be used to address the objectives: (1) to estimate the genetic correlations between MCB resistance and agronomic traits; (2) to identify and characterize QTLs responsible for MCB resistance and agronomic traits within the European Flint group.

## Results

EP42 had higher stalk tunnel length than EP39, while EP39 × EP42 had a value close to the mid-parent value (Table 1). In contrast to stem resistance, EP39 × EP42 had higher level of ear resistance than the most resistance parent. The average value of the RILs was close to the mid-parent value for several traits, either agronomic or related to resistance. Genetic variances in the RIL population were highly significant for all traits, while the genotype × environment interaction variances were highly significant for agronomic traits, but not for resistance traits, except for cob damage.

**Table 1:** Characteristics of EP39 and EP42, EP42 × EP39 and the RIL population developed from EP42 × EP39.

	Means				Variances		
	
	EP39	EP42	F1	RILs			
	
Stalk tunnel length (cm)	19.9 ± 5.5	54.9 ± 5.5	43.4 ± 4.0	38.9 ± 4.0	39.3 ± 8.0**	11.8 ± 6.6	101.6 ± 6.3**
Relative stalk tunnel length	0.38 ± 0.11	0.51 ± 0.11	0.32 ± 0.03	0.42 ± 0.07	0.0016 ± 0.0006**	0.0013 ± 0.0007	0.01125 ± 0.0007**

Kernel damage (1-9 scale)^a^	6.67 ± 0.58	6.82 ± 0.59	8.43 ± 0.16	7.38 ± 0.12	0.098 ± 0.0557	0.080 ± 0.070	1.148 ± 0.073**

Shank damage (1-9 scale)^a^	3.09 ± 0.37	3.90 ± 0.37	5.97 ± 0.69	4.05 ± 0.43	0.55 ± 0.17**	0.00 ± 0.00	2.68 ± 0.20**

Cob damage (1-9 scale)^a^	5.33 ± 0.35	7.37 ± 0.35	8.50 ± 0.10	7.53 ± 0.21	0.632 ± 0.123**	0.041 ± 0.003**	1.217 ± 0.093**

Anthesis (days)	64.6 ± 7.4	66.3 ± 7.4	59.2 ± 6.1	66.8 ± 7.2	7.88 ± 1.02**	1.23 ± 0.35**	4.23 ± 0.26**

Silking (days)	67.3 ± 6.5	67.6 ± 6.5	61.3 ± 5.7	68.8 ± 6.3	9.05 ± 1.25**	2.31 ± 0.52**	5.26 ± 0.32**

Plant height (cm)	56.4 ± 4.0	109.7 ± 4.0	132.5 ± 7.8	93.7 ± 4.2	121.2 ± 18.0**	26.5 ± 9.6**	126.8 ± 7.9**

Grain humidity (%)	18.9 ± 0.7	15.5 ± 0.7	19.4 ± 0.3	17.8 ± 0.3	0.82 ± 0.33**	1.59 ± 0.35**	3.46 ± 0.21**

Yield (t ha^-1^)	0.93 ± 0.25	2.51 ± 0.25	6.67 ± 0.96	1.80 ± 0.24	0.37 ± 0.08**	0.26 ± 0.06**	0.64 ± 0.04**

Estimates of means ± standard deviation, as well as of variance components were obtained for several agronomic and resistance traits after evaluation in two environments under artificial infestation with corn borer eggs

^a ^Kernel, Shank and cob damages were estimated on a 9 point scale (9 = without injury; 1 = wholly damaged)

** Variance component was significant at the 0.01 probability level

Regarding the agronomic traits, a negative genetic correlation coefficient between flowering and yield was found among RILs, while a positive genetic correlation coefficient was found between flowering and grain humidity (Table 2). Regarding the relationship between agronomic and resistant traits, plant height and flowering had a positive and moderate genetic relationship with stalk tunnel length.

**Table 2:** Phenotypic and genetic correlation coefficients among resistance and agronomic traits calculated in the population of RILs derived from EP39 × EP42.

	AT^a^	S	PH	H	Y	STL	RSTL	SDR	CDR
**AT**		0.92*	0.01	0.32*	-0.36*	0.10*	0.11*	-0.11*	-0.21*
**S**	0.97^+^		-0.01	0.32*	-0.42*	0.11*	0.13*	-0.12*	-0.20*
**PH**	0.23^+^	0.20		0.07	0.37*	0.48*	-0.05	0.15*	0.05
**H**	0.85^+^	0.74^+^	0.53		-0.14*	0.07	0.05	0.01	-0.20*
**Y**	-0.68^+^	-0.68^+^	0.27	-0.84		0.12*	-0.07	0.17*	0.16
**STL**	0.32^+^	0.43^+^	0.51^+^	0.61	-0.03		0.82*	-0.25*	-0.18
**RSTL**	0.19	0.38^+^	-0.28	0.29	-0.35	0.70^+^		-0.37*	-0.24*
**SDR**	-0.44	-0.53^+^	0.12	-	-0.19	-0.08	-0.09		0.51*
**CDR**	-0.76	-0.41	0.56	-	0.22	0.94	0.58	0.68^+^	

The phenotypic correlation coefficients are shown above the diagonal while the genetic correlation coefficients are shown below the diagonal. The correlation coefficients were obtained after evaluation in two environments under artificial selection with corn borer eggs

^a ^AT = anthesis, S = silking, PH = plant height, H = grain humidity, Y = yield, STL = stalk tunnel length, RSTL = relative stalk tunnel length, SDR = shank damage rating, CDR = cob damage rating.

* Phenotypic correlation was significant at the 0.01 probability level.

^+ ^Genotypic correlation exceeded twice its standard error.

**Figure 1:**
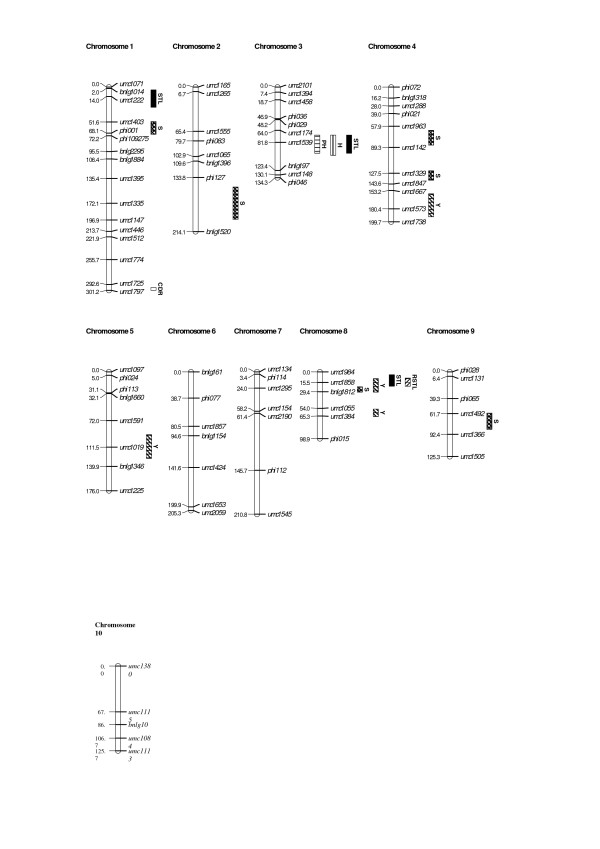
**Molecular linkage map and location of the QTLs detected in the RILs population derived from EP42 × EP39**. S = silking, PH = plant height, H = grain humidity, Y = yield, STL = stalk tunnel length, RSTL = relative stalk tunnel length, CDR = cob damage rating.

The genetic map had a total length of 1791 cM and an average distance between loci of about 20 cM (Figure 1). In the RILs, three QTLs were detected for stalk tunnel length at chromosomes 1, 3 and 8 (Figure 1; Table 3). Those QTLs explained 33% of the genetic variance, calculated with the whole data set, but this value was reduced to 7.5% when the cross validation method was used. The additive values for the QTLs varied between 2.4 and 2.8 cm and both parents contributed with favourable alleles. The QTL at bin 8.05 was also related to relative stalk tunnel length. Concerning ear resistance traits, one QTL was detected for cob damage rate with a LOD score lower than 3 and a validation frequency lower than 30%.

**Table 3:** Summary of QTLs detected in the RIL population derived from a EP39 × EP42 evaluated in two environments under artificial infestation with corn borer eggs.

							Cross validation â_TS.ES_^d^			
							
QTL bin^a^	Confidence interval	LOD score	Flanking markers	R^2^_adj_	*p*^b^	â^c^	Median	Percentile (10, 90)	Frequency (%)	P
**Stalk tunnel length (cm)**										
1.02	4-30	4.25	bnlg1014 umc1222	11.6		-2.83	-2.85	(-3.33, -2.42)	91.9	
3.05	71-98	3.87	umc1174 umc1539	9.6		2.40	2.47	(2.14, 3.01)	78.7	
8.05	4-21	3.46	umc1984 umc1858	9.6		2.57	2.63	(2.22, 3.22)	77.7	
*Final fit*					33.2					8.7
**Relative stalk tunnel length**										
8.05	9-21	4.91	umc1984 umc1858	15.0		0.02	0.02	(0.018, 0.027)	65.0	
*Final fit*					28.4					0.0
**Cob damage (1-9 scale)^e^**										
1.12	297-301	2.54	umc1725 umc1797	7.5		0.29	0.33	(0.29, 0.37)	29.0	
*Final fit*					7.0					0.0
**Silking (days)**										
1.03	51-70	3.22	umc1003 phi001	9.5		-0.94	-0.98	(-1.19, -0.85)	34.9	
2.08	148-196	3.48	phi127 bnlg1520	11.2		2.11	2.19	(1.86, 2.63)	50.4	
4.04	64-86	4.87	umc1963 umc1142	13.7		1.58	1.43	(1.19, 1.71)	55.3	
4.06	124-138	2.80	umc1329 umc1847	8.2		-0.87	-1.07	(-1.29, -0.91)	59.7	
8.05	22-30	10.8	umc1858 bnlg1812	29.4		-1.62	-1.41	(-1.64, -1.13)	92.1	
9.05	61-85	5.49	umc1492 bnlg1812	15.9		1.35	1.27	(1.06, 1.55)	63.3	
*Final fit*					45.5					18.9
**Plant height (cm)**										
3.05	71-98	5.97	umc1174 umc1539	16.6		5.04	5.27	(4.18, 6.94)	98.4	
*Final fit*					16.1					12.8
**Grain humidity (%)**										
3.05	71-101	5.60	umc1539 bnlg197	16.0		0.63	0.66	(0.51, 0.89)	95.8	
*Final fit*					11.7					0.7
**Yield (t ha^-1^)**										
4.08	158-192	2.44	umc1667 umc1573	7.3		0.21	0.22	(0.18, 0.26)	36.5	
5.05	93-128	2.85	umc1591 umc1019	8.5		-0.18	-0.22	(-0.28, -0.19)	44.6	
8.05	10-30	3.46	umc1858 bnlg1821	10.3		0.23	0.23	(0.20, 0.28)	39.8	
8.08	55-66	4.07	umc1055 umc1384	12.5		0.25	0.24	(0.20, 0.32)	51.4	
*Final fit*					22.5					9.06

^a ^Bin locations are designed by an X.Y code, where X is the linkage group containing the Bin and Y is the location of the Bin within the linkage group (Gardiner et al., 1993).

^b ^Proportion of the genotypic variance explained by detected QTL, adjusted for QTL × Environment interaction, and calculated using the whole data set.

^c ^Additive effects calculated in the whole data set. Positive additive effects indicate that the EP42 allele increases the value of the trait.

^d ^Median and percentiles of the additive effects, frequency of QTL detection and proportion of the genotypic variance explained by detected QTL alculated in 200 cross-validation runs.

^e ^Cob damage was estimated on a 9 point scale (9 = without injury; 1 = wholly damaged)

In the RILs, we also detected 6, 1, 1, and 4 QTLs for silking, plant height, kernel humidity, and yield, respectively (Figure 1; Table 3). For these traits, the percentage of genetic variance explained by the QTLs, calculated in the whole data set, varied from 10 to 45%. However, the percentages of genetic variance explained by the QTLs were much lower when they were calculated by cross validation. The QTL for silking at bin 8.05 is remarkable because it explains 29% of the phenotypic variance. This QTL was detected for silking in the 92% of the CV/G runs, indicating a great precision in the location. A QTL for yield at bin 8.05 was located near a QTL related to stalk tunnel length and relative stalk tunnel length. A QTL for plant height and grain humidity was found at bin 3.05 and explained 16% of the phenotypic variance, approximately, for each trait. For both traits, the QTL was detected with great precision (more than 95% of the CV/G runs). For silking, each parent contributed with favourable alleles to half of the QTLs, while for yield, the favourable alleles for most QTLs came from EP42. EP42 also provided the allele that increased the trait for the QTL related to plant height and kernel humidity.

## Discussion

As expected from previous experiments [29,30] EP39 was resistant, while EP42 was susceptible to MCB stalk tunnelling. The value of EP39 × EP42 for stalk tunnel length was close to the mid-parent value which suggests that additive effects were more important than dominant effects. This is in concordance with previous experiments in which the dominant effects were not significant, except for one single cross [14-16]. Contrarily to stalk tunnel length, the hybrid was more ear resistant than the most resistant line indicating that dominant effects would also play an important role, in agreement with results reported by Cartea et al. [18] and Velasco et al. [31]. Similarly, the EP39 × EP42 hybrid exhibited a considerable degree of heterosis for most agronomic traits. Although both parental lines did not differ for many traits, the significant genetic variation found among RILs for all traits, except for kernel damage, showed that this population is a valuable material to detect QTLs among European germplasm, especially for MCB resistance. The lack of significant RILs × environment interaction for stalk tunnel length is in agreement with previous research [10,29,32].

Plant height was highly and significantly correlated to a resistance trait, such as stalk tunnel length. This result is in agreement with the result reported by Schon et al. [33], but not with results obtained by other authors [7,9,34]. This suggests that the genetic relationship between both traits depends on the germplasm being evaluated.

The 3 QTLs for stalk tunnelling by MCB detected in this study did not overlap with the QTLs for stalk tunnelling by MCB detected in the intermated B73 × Mo17 population [10]. Differences due to genetic heterogeneity or sampling limited number of progeny could explain the lack of coincidence between two particular QTL experiments. On the contrary, the QTLs for stalk tunnelling detected in the present study were in the same or adjacent bins to QTLs for stalk tunnelling by ECB consistently detected in other experiments [7-9,33-35]. The coincidence of the three QTL locations in experiments carried out with genetically diverse maize populations and with different corn borer species indicates the importance of those genomic regions for corn borer resistance across corn borer species and maize populations. The resistance mechanisms of maize to ECB or MCB attack at early stages of plant development are probably based on toxins, for example DIMBOA, but, based on structural compounds, particularly cell wall composition, later on [36-39]. Cell wall characteristics may affect insect feeding due to different reasons: elevated levels of indigestible fiber may increase the bulk density of the diet to the point that insect are unable to ingest sufficient quantities of nutrients and water [40], and/or lignified cell walls may produce tougher tissues that are more resistant to the tearing action of mandibles [41]. QTLs for MCB stalk tunnelling detected in this experiment at bins 1.02, 3.05, and 8.04 were close to QTLs for stalk strength or cell wall compounds detected in other experiments, suggesting that genes involved in the synthesis of cell wall compounds in maize could be good candidate genes for resistance to corn borers. Thus, Flint-Garcia et al. [42] detected only one QTL for stalk strength in common across four populations which is approximately located in bin 3.05. Regarding cell wall main components, QTLs for neutral detergent fiber (NDF), acid detergent fiber (ADF), acid detergent lignin (ADL) and hemicellulose were detected in bins 1.01/1.02, 3.05/3.06 and 8.03/8.04 [43-47]. In addition, Barriere et al. [43] found cell wall-bound phenolic compounds (p-coumaric acid, esterified ferulic acid, etc) to be associated to the three genomic regions, particularly to bin 1.01/1.02. Silage corn digestibility for dairy cattle is related to cell wall characteristics [48] and therefore probably related to maize resistance to corn borers too. Thus, QTLs for silage corn digestibility were also detected in bins 1.01/1.02 and 3.05/3.06 [43,49]. Furthermore, out of the five expression QTL (eQTL) hotspots for silage corn digestibility detected by Shi et al. [50], the two main ones were in bins 8.03 and 3.05. The eQTL hotspot on bin 3.05 was co-localized with a QTL for cell wall digestibility, concluding the authors that the gene underlying QTL and eQTL are identical.

In the region of bin 3.05 approximately 1000 protein-coding genes of rice and sorghum aligned to maize genome http://www.plantgdb.org/ZmGDB/DisplayGeneAnn.php?ds=&q=. For that reason, the isogenization-assisted by molecular markers - of the QTL could narrow its interval and facilitate the clonation of genes. However, we propose some candidate genes according to the hypothesis that genes involved in cell wall biosynthesis or fortification confer also resistance to corn borers. Candidate genes for stem tunnelling were selected from the gene expression data repertory of cell wall biosynthesis and assembly in maize contained in MAIZEWALL http://www.polebio.scsv.ups-tlse.fr/MAIZEWALL/index.html, the genes located in those QTL regions. We have found in the region 3.05 three genes from the phenyhlpropanoid pathway which is the pathway that controls the biosynthesis of monolignols, the monomers of lignins [51]. These genes are: a peroxidase (*GRMZM2G103342*, ctg126, AC211202: 70944-73152, http://www.maizesequence.org/Zea_mays2/geneview?db=core;gene=GRMZM2G103342), a laccase (*GRMZM2G072780*, ctg137, AC207620: 82019-85418, http://www.maizesequence.org/Zea_mays2/geneview?db=core;gene=GRMZM2G072780), and a p-coumarate-3-hydroxylase (C3H) (*GRMZM2G138074*, ctg138, AC200558: 57765-60725, http://www.maizesequence.org/Zea_mays2/geneview?db=core;gene=GRMZM2G138074). In addition to the MAIZEWALL repertory of genes, we also searched in MaizeGDB for genes related to cell wall biosynthesis located at the QTLs' regions. In bin 3.05 lies a gene that codifies for the sucrose phosphate synthase 2 enzyme (ctg 131, between position 152605600 and 152708500, http://www.maizegdb.org/cgi-bin/displaylocusrecord.cgi?id=96665) which is involved in cellulose biosynthesis [52]. The *lax midrib1 *gene which affects the midrib portion of the leaf [53] is also in the region (ctg 132, between position 161945000 and 163130800, http://www.maizegdb.org/cgi-bin/displaylocusrecord.cgi?id=12405). Inbred lines of maize with lax midribs have lower levels of fiber, lignin and xylose and are more digestible than 'normal' inbreds [54]. The five genes at bin 3.05 constitute possible candidate genes for resistance to stalk tunneling that could be validated by an association study [55].

For stalk tunnel length the proportion of genotypic variance explained by the QTLs following cross validation in our experiment was similar to that found by Papst et al. [34] and Ordas et al. [10]. Given the low number of detected QTL and the small proportion of genotypic variance explained, it is likely that the trait be regulated by many QTL of small effect. Therefore, according to different QTL experiments, for resistance to corn borer tunnelling the theoretical expectation of the efficacy of MAS for increasing resistance to corn borers is low and it can be concluded that MAS seems not promising. However, the genomic regions related to resistance to corn borer detected in this and others QTL experiments are useful as start points for fine mapping in order to address, in the future, the cloning of genes related to resistance. Regarding the utility of the QTLs for maize breeding, we evaluated a sample of 118 of the RILs crossed to a tester (A641) in two different sowing dates in the same location and year (data not shown), and no QTLs for resistance were found in the testcross population.

We found, consistently with a previous experiment with MCB [10], that MCB produce higher tunnels length than ECB [7,9,35]. In both experiments with MCB we found less QTLs for stalk tunnelling, 3 and 2 respectively, than the average number of QTLs reported for ECB stalk tunneling that ranged from six to nine [7,8,33-35]. As argued by Ordas et al. [10], it is possible that, due to the aggressiveness of the insect, most genotypes seem to be susceptible, although some of them carry a low level of resistance. This is in agreement with the phenotypic performance of the two parents and the segregation among RILs.

Regarding the agronomic traits, the major QTL for flowering time detected at bin 8.05 is located within a consensus region of major effect [56] with at least two different QTLs: *vgt1*, recently cloned by Salvi et al. [57] and *vgt2 *[58]. The confidence interval of this QTL for flowering time overlapped with the confidence interval of a QTL for yield. As the allele that increased flowering time decreased yield, the co-localization of the two QTLs could partially explain the significant and negative genetic correlations between flowering time and yield that we found. The QTL for plant height at bin 3.05 was consistently found in different genetic backgrounds and environments [7,33,59-62]. The confidence interval of this QTL overlapped with the confidence interval of the QTL for stalk tunnel length, agreeing with a previous experiment [33]. Furthermore, in both experiments the allele associated to increased damage was also associated to increased plant height. The co-localization of the two QTLs, for plant height and stalk tunnel length, could contribute to the positive genetic correlation between both traits detected in this experiment. The confidence interval of the QTL for yield at bin 8.05 overlapped with the confidence interval of a QTL for stalk tunnel length and relative stalk tunnel length. Both the allele for decreased yield and the allele for increased stalk resistance were provided by the same line and, therefore, if the QTL would be used for increasing resistance to corn borers by MAS, a negative effect on yield could be expected. A negative relationship between resistance and yield was also found in selection programs in which the yield decreased as an indirect consequence of selecting for increased resistance [19-21]. However, it is not possible to know if the co-localization of QTLs for different traits is due to linkage between different genes or pleitropism of a single gene.

## Conclusions

We detected three genomic regions involved in resistance to stalk tunnelling by MCB that were close to genomic regions related to resistance to stalk tunnelling by ECB detected in genetically different populations. This indicates the importance of those genomic regions across corn borer species and maize populations. Our results, when compared with results from other authors, suggest that genes involved in cell wall biosynthesis or fortification could be good candidate genes for the QTLs detected for stem tunnelling in our experiment. Particularly, we proposed five candidate genes related to cell wall characteristics which could explain the QTL for stalk tunnelling at bin 3.05. The small proportion of genotypic variance explained by the QTLs suggest that there are also many other genes of small effect regulating stem tunnelling by MCB. Therefore, we conclude that MAS seems not promising for this trait, although the genomic regions consistently detected are useful as starting points for the cloning of genes related to resistance. Two of the QTLs detected for stalk tunnelling overlap with QTLs for agronomic traits, indicating the presence of pleitropism or linkage between genes.

## Methods

### Plant materials

We developed a population of 178 RILs from the cross EP42 × EP39 by single-seed descent. EP42 is a yellow European flint line that was obtained from a local open pollinated variety from North-Western Spain (humid Spain), while EP39 is a yellow European flint line that was obtained from the race 'Fino' from Central Spain (dry Spain). EP42 is susceptible to MCB tunnelling, while EP39 is resistant to MCB attack [29,30]. The seed of the RILs was obtained by hand pollination in Northwestern Spain in 2005.

### Phenotypic analysis

The parental inbred lines, the RILs, and EP39 × EP42 were evaluated in 2006 and 2007 in Pontevedra (42° 30'N, 8° 46'W), located in Northwestern Spain at the sea level, on the Atlantic coast. In Pontevedra, temperatures are relatively mild all year and the average annual rainfall is around 1700 mm. The evaluation was carried out under artificial infestation with corn borer eggs. At each environment, the treatments were arranged in a 16 × 12 α-lattice design with three replications per environment. For the evaluation of the RILs each plot consisted of one row with 13 hills per plot, rows were spaced 0.80 m apart and hills were spaced 0.21 m apart. Plots were overplanted and thinned obtaining a final density of approximately 60 000 plants ha^-1^. The seedbed preparation was made according to the standard practices of the area: a chisel plow followed by a rotary tiller. Prior to emergence a pre-emergence herbicide was applied. When the plants were about 60 cm tall, later weeds were controlled by cultivation with a shovel cultivator. Fertilization was made with 105 Kg of N, 105 Kg of P_2_O_5_, and 105 Kg of K_2_O. Prior to flowering we applied 55 additional Kg of N. We irrigated with 60 L m^-2 ^at flowering.

For each plot, the date of silking was considered when 50% of the plants of the plot exerted the silks from within the husks. At silking, five plants for each plot were infested with a mass of ≈ 40 eggs of corn borer which were placed between the main ear and the stem [63]. At harvest, stems of the infested plants from each plot were dissected, the total tunnel length (cm) of each plant measured and the corn borer tunnelling reported both in centimetres (stalk tunnel length) and as ratio of tunnel length and plant height (relative stalk tunnel length). Kernel damage was estimated on a 9 point scale (9 = without injury; 1 = wholly damaged). The following agronomic traits were also taken: the number of days from the date of planting to the date of anthesis, plant height, grain humidity at harvest (%), and yield at 140 g kg^-1 ^moisture content. In addition to the previous traits, shank and cob damage ratings (9 = without injury; 1 = wholly damaged) were estimated for each plot.

Individual analyses of variance and adjusted means were calculated for all traits according to a α-lattice design using the Mixed Procedure of SAS [64]. Combined analysis of variance over years was computed using the adjusted means. Variance components were estimated by restricted maximum likelihood (REML). Computations were performed with SAS [64]. Phenotypic (r_p_) and genetic (r_g_) correlations between traits were estimated with a multivariate REML procedure following Holland [65] and using the SAS programs developed by the author http://www4.ncsu.edu/~jholland/correlation/correlation.html.

### QTL analysis

DNA of ten plants picked at random from each RIL was extracted according to Liu and Whittier [66] with modifications. SSR amplifications were performed as described by Butron et al. [67]. SSR products were separated after amplification by electrophoresis using 1 TBE on a 6% non-denaturing acrylamide gel (approximately 250 V for 3 h) [68]. Two hundred twenty six SSR primers pairs were used to genotype the RILs. From these, eighty four SSR that were polymorphic and give clear bands patterns were used for linkage mapping and QTL analysis, resulting in a uniform distribution of markers along the genome. The linkage map was built using MAPMAKER 3.0b [69]. Loci were assigned to linkage groups which were anchored to chromosomes using default parameters (minimum LOD of 3.00, maximum distance of 30 cM and maximum unlinked LOD of 2.00). Multipoint linkage analysis was performed for each linkage group by the "order" command using an informativeness criteria of 100 individual and a distance between markers of 2.00 cM. Charts of chromosomes and QTLs were obtained by using MapChart [70]. Composite interval mapping [71] was conducted with PLABQTL [72] with cofactor selection performed following PLABQTL's recommendations and using an "*F*-to-enter" and an "*F*-to-delete" value of 7. A LOD threshold of 2.4 was determined by permutation tests that ensures an experiment wise error rate of p < 0.20. A simultaneous fit with the detected QTLs was performed for each environment and a QTL ANOVA was carried out with PLABQTL [73]. The mean squares of the ANOVA were used to obtain an estimate of the proportion of the genetic variante explained by the detected QTL which is adjusted by QTL × environment interaction [73].

Fivefold cross validation (CV/G) was performed, following the procedures described by Utz et al. [74], to estimate the additive effects and the proportion of genotypic variance explained by the QTLs. The whole data set was randomly split into *k *= 5 data subsets. Four of these subsets were combined to form the estimation set (ES) and the remaining subset formed the test set (TS) in which predictions derived from ES were tested for their validity by correlating predicted and observed data. We used 1000 replicated CV/G runs. For a particular QTL and its confidence interval estimated using the whole data set, the frequency of QTL detection across the CV/G runs was calculated by counting the number of CV/G runs in which a QTL was located within that confidence interval. The frequency of QTL detection gives us an estimation of the precision of QTL localization [73].

## Authors' contributions

BO carried out field experiments, performed data analysis and drafted the manuscript. RAM conceived the study, participated in its design and in its coordination. RS assisted BO in field experiments. AB conceived the study, participated in its design, carried out molecular analysis and assisted BO in data analysis. All authors read and approved the final manuscript.
